# Tracking of obesity among 2‐ to 9‐year‐olds in an electronic heath record database from 2006 to 2018

**DOI:** 10.1002/osp4.407

**Published:** 2020-02-08

**Authors:** David S. Freedman, Alyson B. Goodman, Raymond J. King, Heidi M. Blanck

**Affiliations:** ^1^ Division of Nutrition, Physical Activity and Obesity Centers for Disease Control and Prevention Atlanta Georgia

**Keywords:** BMI, child obesity, databases, longitudinal

## Abstract

**Background and Objective:**

As obesity among children and adolescents is associated with major health risks, including the persistence of obesity into adulthood, there has been interest in targeting prevention efforts at children and adolescent. The longitudinal tracking of BMI and obesity, as well as the effects of initial age and duration of follow‐up on this tracking, were examined in a large electronic health record (EHR) database.

**Methods:**

The data consisted of 2.04 million children who were examined from 2006 through 2018. These children were initially examined between ages 2 and 9 years and had a final examination, on average, 4 years later.

**Results:**

Overall, children with obesity at one examination were 7.7 times more likely to have obesity at a subsequent examination than children with a BMI ≤ 95^th^ percentile. Further, 71% of children with obesity at one examination continued to have obesity at re‐examination. Although 2‐year‐olds had a relative risk of 5.5 and a positive predictive value of 54%, then sensitivity of obesity at younger ages was low. Of the children who were re‐examined after age 10 y and found to have obesity, only 22% had a BMI ≥ 95^th^ percentile at age 2 years.

**Conclusions:**

Despite the tracking of obesity at all ages, these results agree with previous reports that have found that an elevated BMI at a very young age will identify only a small proportion of older children with obesity.

**Abbreviations**
BMI: body mass index
CDC: Centers for Disease Control and Prevention
BMIz: BMI‐for‐age z‐score
L: power transformation for normality
M: median
S: coefficient of variation

## 1 INTRODUCTION

As almost 40% of adults in the United States have obesity,^1^ many prevention efforts have focused on children and adolescents. The tracking of BMI levels and obesity among children has been well documented,^2^, ^3^, ^4^, ^5^ and in general, children with a high BMI at one examination are likely to have a high BMI upon re‐examination in adolescence and adulthood. However, the strength of this association varies substantially by the age of the child at the initial examination and the time interval between examinations.^2^, ^5^

Although a high BMI before age 5 years is also predictive of a subsequently elevated BMI,^6^ the importance of a high BMI in among preschool children has been questioned.^7^, ^8^, ^9^, ^10^ For example, only about 20% (sensitivity) of children with obesity at age 5 years had a rapid weight gain between birth and 2 years.^7^ Wright *et al*.^8^ also reported a low sensitivity for rapid weight gain in infancy, with most 8‐year‐olds with obesity having been normal‐weight infants. Further, von Kries *et al*.^9^, ^10^ have shown that a large proportion of 6‐year‐olds with obesity were in the normal‐weight category at age 2 years. However, since the prevalence of obesity among very young children is relatively low, several of these estimates have been based on fairly small numbers.

The objectives of the current analyses are to (1) examine the tracking of BMI levels among children and adolescents, and (2) to assess the ability of a high BMI at one examination to predict a subsequently elevated BMI. The sample is based on a large electronic health record (EHR) database of 2.04 million children who were initially examined between ages 2 and 9 years and were re‐examined, on average, 4 years (range, 1 to 13 years) later. Analyses focus on the effects of age at the first examination and the time interval between examinations on the tracking of obesity.

## 2 SUBJECTS AND METHODS

### 2.1 Study sample

IQVIA's Ambulatory Electronic Medical Record (AEMR‐US) database contains de‐identified medical records and encounter from 44,000 physicians and 315 networks in the United States covering the period from January 2006 through May 2019. These data include provider medical specialty, patient variables such as examination data, year of birth, gender, race and ethnicity, and clinical variables such as diagnoses, procedures, medication prescriptions records, and patient and family history captured during a patient visit. Contributing practices consist of medium to large physician offices, outpatient clinics, and physician groups.

Using the E360 Software‐as‐a‐Service (SaaS) Platform,^11^ 5.6 million 2‐ to 19‐year‐olds with longitudinal data for weight and height (33 million records) were identified in the AEMR‐US database. Because examination date and year of birth, but not age, were available, age was estimated from the examination date and the midpoint of the birth year. About 90% of the weights and heights were recorded in US standard units and were converted to metric units.

For these secondary analyses of these data, it was required that (a) there was at least one year between the initial and final examinations and (b) that the first examination occurred before age 10 years resulting in 2.2 million children. A total of 454 children (56,000 records) examined more than 100 times during this 12‐year period were excluded from the analysis as extensive healthcare usage may suggest a chronic condition

Weight and height were coded in pounds and inches for 90% of these records, and in kg and cm for 6% of the records. Preliminary analyses indicated that of the 667,000 records that had weights and heights in mismatched units (e.g. weight in kg and height in inches), many of the weights coded as kg were actually recorded in pounds. Because of these coding errors, the analyses are limited to the 16.2 million records (2.14 million children) that had consistent units for both weight and height.

Weight and height values that were likely to be errors were identified and excluded using Daymont's algorithm for the longitudinal detection of outliers.^12^, ^13^ This method focuses on the difference between modified z‐scores for weight and height^14^ at each examination and the expected values based on an exponentially weighted moving average for each child. This method also identifies other categories of potential errors, such as a height decrease of > 3 cm between examinations, and values that are carried forward from a previous examination. For children with several weights or heights on the same date, the algorithm also indicates which measurement is most consistent with other values for the child.

Based on this algorithm, 785,000 weights that were identical (carried forward) to that at the previous examination were excluded. Of the 2.6 million heights that were identified as carried forward, 1.5 million were excluded if they either (a) followed a height that was a flagged as a likely error or (b) occurred among younger subjects (boys <17 years, girls <16 years) and remained the same for more than 3 months. It was thought that carried forward heights among older children could represent attained, adult height. Excluding these potential errors identified by the Daymont algorithm and reapplying the age and follow‐up restrictions reduced the number of children from 2.14 million to 2.04 million. The CDC cut‐points for implausible values^14^ were not used in the current study as there were many extremely high weights and BMIs that were consistent across examinations. However, 41 records that had a modified BMIz ≥25 or a BMI ≥ 150 were excluded.

There appeared to be some potential unit errors remaining for weight, and Friedman's super smoother^15^, ^16^ was used to examine the distance between each child's weight and its smoothed value. After inspection of these plots, an additional 126 children who had a residual that was above the 99.95^th^ percentile of the distribution and a weight more than 10 kg from its smoothed value. This resulted in a dataset of 2,036,015 children (13,347,608 records). Most analyses focused on the only first and last examination for each child.

### 2.2 BMI metrics

BMI‐for‐age z‐scores (BMIz) and percentiles were calculated from the CDC growth charts^17^, ^18^ using sex‐ and age‐specific values of L (power transformation to achieve normality), M (median) and S (coefficient of variation)^19^, ^20^:

$$\text{BMIz}=\frac{{\left(\mathrm{BMI}/M\right)}^{L}-1}{L\times S}\;$$

Because the values of L parameter in the CDC growth charts are much less than −1.0 at most ages^21^ and because the estimation of L, M and S were not based on BMIs above the 97^th^ percentile,^22^ very high BMIs are compressed into a narrow range of z‐scores and do not correspond well with the observed data.^23^, ^24^, ^25^, ^26^ However, since the analyses focus on obesity, defined as a BMI ≥ 95^th^ percentile of the CDC growth charts, it was decided not to use other BMI metrics that have been proposed.^27^ Severe obesity was defined as a BMI ≥ 120% of the 95^th^ percentile.^28^

### 2.3 Statistical methods

The primary focus is on the cross‐classification of the obesity status of the 2.04 million children at the first and last examinations, and the relative risks (RR), sensitivities, specificities and positive predictive values were assessed. Stratified analyses were performed to assess the influence of the initial age of the child and the time interval between the two examinations on this association. Additional analyses examined the consistency of obesity across examinations among children who had measurements at three ages: before 4 years, between 6 and 8 years, and after 10 years. Given the large sample size, only a few confidence intervals are presented because all were very narrow.

The prevalence of obesity in this EHR database was also compared with the corresponding estimates in the National Health and Nutrition Examination Survey (NHANES) for each year and age group. For this comparison, one BMI value was randomly selected within each combination of age group and year of study from a child.

Data management and analyses were performed in R.^29^

## 3 RESULTS

**Table** 1 shows descriptive characteristics of the children at the first and last examinations. The mean ages were 5.0 ± 2.5 years (first examination) and 9.0 ± 3.8 years (last examination), and the time interval between the examinations varied from 1 to 13 years (mean 4 years). Mean BMI increased by 2 kg/m^2^ between examinations, the prevalence of obesity increased from 13.4% to 17.6%, and the mean BMIz increased from 0.33 to 0.49 as the children aged. Most of the children were white, but the race/ethnicity of about 25% was unknown and only 2.4% were Hispanic.

**Table 1:** Descriptive characteristics at the initial and final examinations among 2 036 015 children and adolescents

	Initial Exam	Final Exam
% girls	48%	
Age (y)^**a**^	5.0 ± 2.5^a^	9.0 ± 3.7
BMI	16.9 ± 2.7	18.9 ± 4.8
BMIz	0.33 ± 1.2	0.49 ± 1.2
Obesity (%)	13.4%	17.6%
Severe obesity (%)	3.0%	5.5%
Race/Ethnicity		
White (%)	60%	‐‐‐
Black (%)	10%	‐‐‐
Hispanic (%)	2.4%	‐‐‐
Asian (%)	3.1%	‐‐‐
Other/unknown (%)	25%	‐‐‐

a For continuous variables, values are mean ± SD.

**Table** 2 shows a cross‐classification of obesity (BMI ≥ 95^th^ percentile) at the first and last examinations. As expected, there was a strong association between obesity (RR = 7.7, 95% CI: 7.72 to 7.73) at the two examinations. Of the children with obesity at the first examination, 71% also had obesity at the final examination (positive predictive value), and about 95% of the children who did not have obesity at the final examination did not have obesity at the first examination (specificity). However, only about 55% (194,953/357,824) of the children with obesity at the final examination had obesity at their first examination (sensitivity).

**Table 2:** Cross‐classification of obesity at the first and last examinations

Obesity at First Exam		Obesity at Final Examination			
		Yes	No	Sum	Classification Statistics
Yes		194 953	78 305	273 258	Relative Risk: 7.7 (7.72, 7.73)^**a**^
No		162 871	1 599 886	1 762 757	Positive Predictive Value: 71.3% (71.2, 71.5)
Sum		357 824	1 678 191	2 036 015	Sensitivity: 54.5% (54.3, 54.6)
					Specificity: 95.3% (95.3, 95.4)

Values are estimate (95% confidence interval).

**Figure 1:**
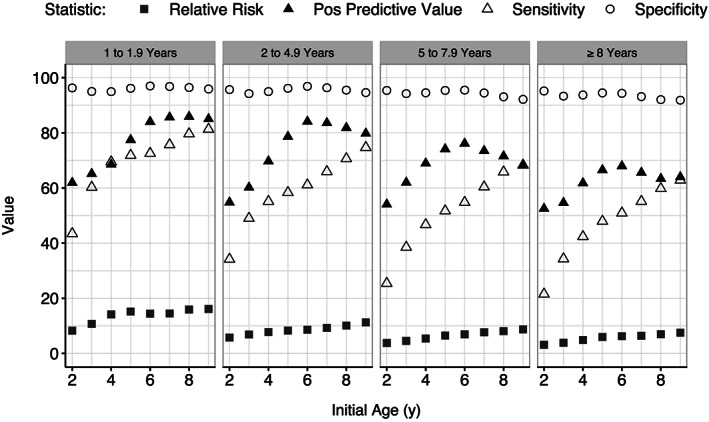
Relation of initial age (x‐axis) and time interval between examinations (four panels) on various statistics from a cross‐classification of obesity at the initial and final examinations

As shown in **Figure** 1, the magnitudes of these statistics varied substantially by both age at the initial examination (x‐axis) and length of follow‐up (four vertical panels panels). For example, among children who were re‐examined from 2 to 4.9 years after the initial examination (second panel), the sensitivity increased with age at the initial examination, from 34% (age 2 years) to 75% (age 9 years). Although the RR also increased from 6 to 11 with initial age in this group, the specificity varied only slightly by age, and the positive predictive value reached a maximum (84%) at about age 6 years. As the time interval between examinations increased (four panels), values of the RR, positive predictive value, and sensitivity decreased. Overall, the percentage of children with obesity at the last examination who had a BMI ≥ 95^th^ percentile at the initial examination (sensitivity) ranged from 22% (initial age 2 years and a follow‐up of ≥ 8 years) to 81% (initial age 9 years with a follow‐up of <2 years).

**Table** 3 shows the consistency of obesity among 87 653 children who were examined at 3 ages, 2 to 3 years, 6 to 7 years, and ≥10 years. Most (74%) of these children did not have obesity at any examination (top row). Of the 8,522 children with obesity at ages 2 to 3 years, 47% had obesity at both subsequent examinations. Among the 17,170 children with obesity after age 10 years (bottom rows), 6374 (37%) children did not have obesity at either of the two previous examinations. The mean BMIz (0.3) at age 2 to 3 years among these 6374 children was close to the mean value(−0.1) among children who did not have obesity at any of the three ages (top row). Further, about one third of these 6,374 children had a BMI at ages 2 to 3 years that was below the CDC sex/age median.

**Table 3:** Obesity status at ages 2 to 3 y, 6 to 7 y, and ≥10 y among 87 653 children

	Obesity Status at Age:			N	Percent^**a**^	Mean BMIz		
	2 to <4 y	6 to <8 y	≥10 y			2 to <4 y	6 to <8 y	≥10 y
Never obese	No	No	No	65,043	74%	−0.1	0	0.1
Obese before age 4 y				8,522				
	Yes	No	No	3,030	36%	2.0	0.8	0.7
	Yes	No	Yes	746	9%	2.1	1.3	1.9
	Yes	Yes	No	736	9%	2.2	2.0	1.2
	Yes	Yes	Yes	4,010	47%	2.5	2.3	2.3
Obese after 10 y				17,170				
	No	No	Yes	6,374	37%	0.3	1.0	1.9
	No	Yes	Yes	6,040	35%	0.6	2.1	2.1
	Yes	No	Yes	746	4%	2.1	1.3	1.9
	Yes	Yes	Yes	4,010	23%	2.5	2.3	2.3

With the exception of the first row (N = 64,807), the denominators for the percentages were either children who had obesity before age 4 y (n = 8,522) or children who had obesity after age 10 y (17 170).

## 4 DISCUSSION

Numerous studies have documented the tracking of BMI and the persistence of obesity throughout childhood.^2^, ^3^, ^4^, ^5^ The current analyses of 2.04 million children showed that as compared with children who had a BMI < 95^th^ percentile, those with obesity at one examination were 7.7 times as likely to have obesity at a subsequent examination. Although 71% of children who had obesity at one examination continued to have obesity at a subsequent examination, the sensitivity was lower, particularly at younger ages. In general, the magnitudes of these statistics increased with the age at the initial measurement and decreased with the time interval between examinations. Among the 10 to 15 years with obesity, only 22% had a BMI ≥ 95^th^ percentile at age 2 years.

The importance of obesity among very young children has been emphasized,^30^, ^31^ and the current results indicate that 2‐year‐olds who have obesity are 5.5 times as likely to have obesity at a subsequent examination than are 2‐year‐olds with lower BMI levels. However, in agreement with other reports, the current results indicate that an elevated BMI at a young age will identify only a small proportion of older children with obesity. For example, it has been reported that 80% of 8‐year‐olds with obesity had not been unusually heavy at 3 months or at 1 year^8^ and that a large proportion of children with obesity at age 6 years had been in the normal‐weight category at age 2 years.^9^, ^10^ The low sensitivity among very young children is consistent across studies despite differences in the ages of the children, the interval between examinations, reference populations and classification of obesity. For example, the magnitudes of the RRs increase at higher BMIs, and the RR for severe obesity (BMI ≥ 120% of the 95^th^ percentile) in the current study was 23 (data not shown).

Estimates of sensitivity can be strongly influenced by differences in prevalence by age, and if the prevalence of obesity increases with age, the maximum sensitivity would be fairly low. In the current study, the prevalence of obesity increased from 8.3% among 2‐year‐olds to 20.5% among children ≥9 years. If these 2‐year‐olds had been re‐examined after age 9 years, the sensitivity could be no higher than 40% (8.3 ÷ 20.5). One possible way to avoid this limitation would be to classify obesity so that its prevalence is constant across ages. Additional analyses, however, based on a BMI ≥ 90^th^ percentile for each sex/age increased the sensitivity of a high BMI at age 2 years for obesity after age 9 years from 23% to only 32%. Furthermore, the mean BMIz (0.3) among the 6374 children who had obesity after age 10 years (Table 3), but not before, were fairly similar to that among children who did not have obesity at any examination in childhood. This indicates that using a lower BMI cut‐point at ages 2 to 3 years, such as the 85^th^ percentile, is unlikely to substantially increase the sensitivity but will reduce the positive predictive value.

There are several limitations in the secondary use of EHR data. The information is not always collected in a standardized way within or across practices, and the view of a patient captured in an EHR is typically representative of a patient's medical history at a single practice or medical group. Therefore, a patient's history in an EHR does not necessarily represent a comprehensive record of all past medical visits. In addition, AEMR‐US data are limited to encounters occurring in specific ambulatory settings.

Although these data are not representative of the US population, the prevalence of obesity in the current study was fairly comparable to estimates from the National Health and Nutritional Examination Survey (NHANES) **(Table** 4).^32^ Although the prevalence estimates between 2011 and 2014 among 2‐ to 5‐year‐olds in these EHR data (11.8%) were higher than those in NHANES (8.4% to 9.4%), the NHANES obesity estimates in 2009 to 2010 and 2015 to 2016 were >12%. The sample sizes in NHANES estimates among 2‐ to 5‐year‐olds ranged from 814 to 903.

**Table 4:** Prevalence of obesity in the longitudinal cohort of children in IQVIA and in four HANES cycles between 2009 to 2010 and 2015 to 2016

Age at Examination (y)		IQVIA^a^		NHANES^b^	
	Examination Year	N	Obesity (%)	N	Obesity (%)
2 to <6	2009‐2010	223 209	11.6 (11.5, 11.7)^c^	903	12.1 (9.8, 14.8)
	2011‐2012	347 166	11.8 (11.7, 11.9)	871	8.4 (5.8, 11.7)
	2013‐2014	511 617	11.8 (11.7, 11.9)	843	9.4 (6.8, 12.6)
	2015‐2016	639 835	11.6 (11.5, 11.7)	814	13.9 (11.6, 16.5)
6 to <12	2009‐2010	227 720	17.4 (17.2, 17.6)	1,213	18.0 (15.9, 20.3)
	2011‐2012	391 415	17.3 (17.2, 17.4)	1,268	17.7 (14.4, 21.5)
	2013‐2014	603 796	17.4 (17.3, 17.5)	1,294	17.4 (13.8, 21.4)
	2015‐2016	773 594	18.0 (18.0, 18.1)	1,268	18.4 (14.9, 22.3)

a Prevalence estimates are based on data from 1 854 490 2‐ to 11‐year‐olds in the longitudinal cohort. For each age group and year combination, one BMI value was selected at random from these children resulting in 3 472 219 BMI values. Obesity is defined as a BMI ≥ 95^th^ percentile in the CDC growth charts.^17^

b NHANES estimates are from Hales *et al.* 32

c Prevalence (95% CI).

Several other limitations of the current study should also be noted. Although efforts were made to exclude data errors,^12^, ^13^ it is likely that errors remained in the data and that some of the excluded values were correct. Although this would have reduced the magnitudes of the observed associations, additional analyses (not shown) indicated that the magnitude of the correlation between initial and final values of BMIz among 5‐ to 9‐year‐olds who were re‐examined 2.5 to 4.9 years later (r = 0.80) was similar to those in the Bogalusa Heart Study.^27^ Furthermore, although information on race/ethnicity was unknown (or ‘other’) for 25% of the children, additional analyses (not shown) indicated that the prevalences of obesity among white (16%), black (23%), and Hispanic (26%) children were also similar to those seen in NHANES 2013‐2016.^33^

## 5 CONCLUSIONS

Despite the limitations of EHR data, the current study indicates that it can be used for a detailed examination of BMI tracking among children. Although there is substantial tracking of BMI from age 2 years, most older children with obesity did not have obesity at age 2 years. This low sensitivity is in agreement with the results of other studies that have showed that most older children with obesity did not have an elevated BMI in early life.

## AUTHORS CONTRIBUTIONS

Dr Freedman conceptualized the study, preformed the analysis, and wrote the initial draft. Dr King performed the data management and critically reviewed the manuscript. Drs Goodman and Blanck critically reviewed the manuscript and were involved in the preparation of the final version.

## CONFLICT OF INTEREST STATEMENT

The authors have no conflict of interest to declare.

## DISCLOSURE INFORMATION

The authors declare no conflict of interest.

## FUNDING INFORMATION

The authors have received no funding for this work.
